# Acute left main coronary artery occlusion with South African flag sign on electrocardiogram: a case report

**DOI:** 10.3389/fcvm.2026.1839575

**Published:** 2026-07-08

**Authors:** Fangming Zhong, Baiqing Lin, Qifeng Zhang

**Affiliations:** 1 Department of Cardiology, Meizhou People’s Hospital, Meizhou, China; 2 Department of Internal Medi I, Pingyuan County People’s Hospital, Meizhou, China

**Keywords:** electrocardiogram, left main coronary artery occlusion, myocardial infarction, percutaneous coronary intervention, South African flag sign

## Abstract

**Introduction:**

The South African flag sign (SAFS) in electrocardiogram (ECG) indicates that the patient may have acute high lateral wall myocardial infarction typically linked to first diagonal branch occlusion, and has rarely been documented in cases of Left Main Coronary Artery (LMCA) occlusion.

**Case presentation:**

A 69-year-old woman with primary hypertension presenting with chest pain for 1.5 h. Initial ECG findings indicated SAFS, suggesting a high lateral myocardial infarction initially believed to involve the first diagonal branch. However, emergent coronary angiography revealed an unexpected complete occlusion of the LMCA with no collateral flow (thrombolysis in myocardial infarction [TIMI] flow grade 0). The patient underwent successful percutaneous coronary intervention (PCI), which restored optimal blood flow (TIMI grade III). The patient was managed with a comprehensive medication regimen, leading to an uneventful recovery and discharge without complications. Follow-up assessments at one and six months showed no adverse cardiac events or symptoms.

**Conclusions:**

This case underscores the need for heightened clinical vigilance when interpreting ECG with SAFS, as it may indicate more severe coronary artery pathology. While a single case cannot redefine diagnostic paradigms, this observation broadens the differential diagnosis of SAFS and reinforces the principle that ECG pattern recognition should always be integrated with clinical context and confirmed by urgent coronary angiography when acute coronary occlusion is suspected.

## Introduction

The South African Flag Sign (SAFS), manifested by ST-segment elevation in leads I, aVL, and V2 with concurrent ST-segment depression in lead III (1–3), has typically been associated with occlusion of the first diagonal branch of the left anterior descending (LAD) artery. While this pattern is a valuable pointer towards the first diagonal branch, its occurrence is not pathognomonic and can occasionally be observed in other anatomic variants or more proximal occlusions, underscoring the complexity of electrocardiographic-coronary anatomical correlations. Nevertheless, the occlusion of the Left Main Coronary Artery (LMCA) commonly displays specific electrocardiogram (ECG) patterns, including widespread ST—segment depression across multiple leads and concurrent ST—segment elevation in leads aVR and V1 (4–6). To date, reports of the South African flag sign (SAFS) in the setting of LMCA occlusion remain exceedingly scarce.

## Case report

### Presenting concerns and clinical findings

**Figure 1:**
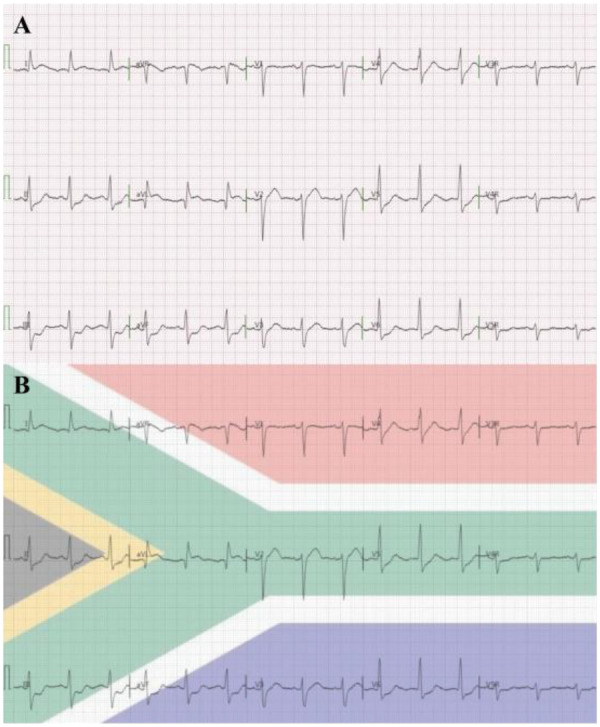
**(A)** the original unmodified 12-lead ECG and **(B)** an annotated version with the South African flag overlay for pedagogical purposes. Figure 1A The patient first electrocardiogram (ECG) in admission in emergency. The ECG revealing ST elevation in leads I, aVL, and V2 with concurrent ST-segment depression in lead III.

A 69-year-old woman with a history of primary hypertension presented to the emergency department with chest pain lasting for 1.5 h. She had no history of diabetes, smoking, alcohol consumption, drug allergies, or surgeries. Upon arrival at the emergency department, her vital signs were as follows: heart rate 86 beats per minute, respiratory rate 20 breaths per minute, SpO2 100%, and blood pressure 120/76 mmHg. Cardiopulmonary auscultation was normal.Initial laboratories drawn on emergency department arrival (∼1.5 h after symptom onset) showed a borderline/marginal high-sensitivity cardiac troponin I (hs-cTnI) of 0.0606 ng/mL (99th percentile upper reference limit [URL]: 0.060 ng/mL), consistent with the early pre-peak phase of troponin kinetics. The acute coronary diagnosis was later confirmed by a marked rise to 9.2 ng/mL at 4 h after reperfusion (see Follow-up and Outcomes). Baseline renal function was preserved, with creatinine 78 µmol/L and estimated glomerular filtration rate (eGFR) 85 mL/min/1.73 m^2^.A 12-lead electrocardiogram (paper speed 25 mm/s; gain 10 mm/mV) demonstrated normal sinus rhythm at 86 beats per minute with a normal QRS axis. There was ST-segment elevation in lead I (1.0 mm), aVL (1.5 mm), and V2 (1.0 mm), accompanied by ST-segment depression in leads II (2.0 mm), III (2.0 mm), and aVF (3.0 mm). Small Q waves (duration <40 ms) were noted in leads I and aVL (Figure 1). Crucially, there was no ST-segment elevation in aVR or V1 and no diffuse ST-depression across the precordial leads—features classically associated with acute LMCA occlusion. No posterior leads (V7–V9) were obtained given the emergent setting. The pattern was consistent with the South African Flag Sign (SAFS), leading to an initial working diagnosis of acute high-lateral-wall ST-elevation myocardial infarction (STEMI) with the first diagonal branch (D1) presumed as the culprit vessel. The patient received 300 mg aspirin and 600 mg clopidogrel.

### Therapeutic intervention

**Figure 2:**
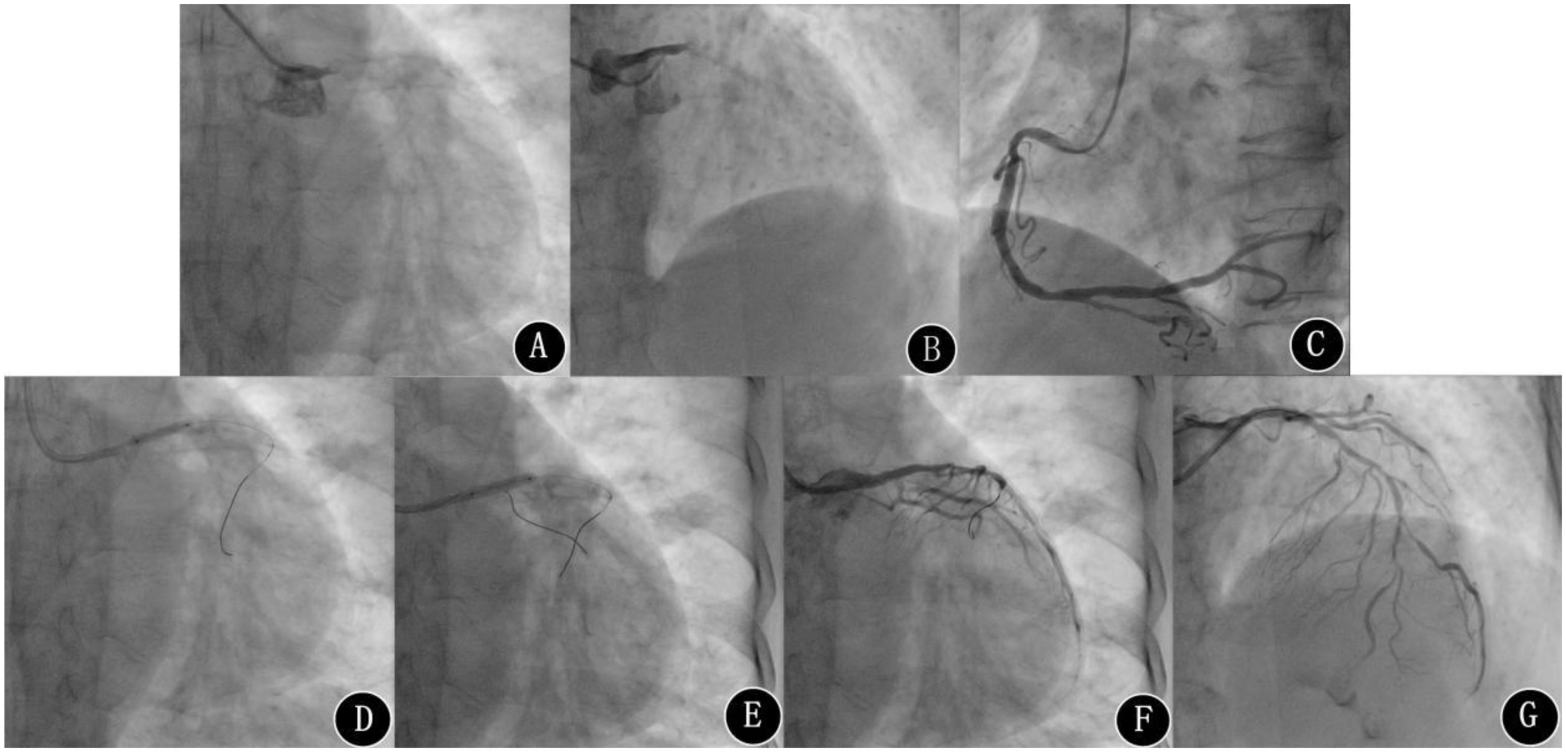
Coronary angiography and intervention. **(A–C)** angiography shows acute occlusion of the left main coronary artery with TIMI flow grade 0. **(D–F)** shows stent insertion in LM-LAD. **(F,G)** is angiography after intervention, and the TIMI flow of the left coronary artery is grade III.

Emergency coronary angiography performed via right radial access revealed a complete occlusion of the left main coronary artery (LMCA) at the body (mid-shaft), with TIMI 0 flow and no visible collateral circulation (Figures 2A–C). Remarkably, the patient remained hemodynamically stable throughout the procedure without signs of cardiogenic shock or ventricular arrhythmias. After engaging the left main with a 6-French EBU 3.5 guiding catheter, a workhorse guidewire was first advanced across the occluded segment into the distal LAD. To protect the circumflex artery, a second guidewire was placed into the distal left circumflex artery (LCX). Pre-dilation was performed with a 2.5 × 15 mm balloon at 18 atm for 5 s (Figure 2D). Since repeat angiography showed minimal residual thrombus burden after balloon dilation, thrombus aspiration was not performed.In such emergent settings, a provisional single-stenting strategy is more commonly employed (accounting for approximately 82% of cases) (7, 8) due to its procedural simplicity, shorter operation time, and lower contrast volume, all of which contribute to faster reperfusion of the ischemic myocardium.A crossover stenting technique was used, placing an EXCROSSAL 3.0 × 14 mm drug-eluting stent (Blue Sail Medical, China) from LMCA to LAD at 18 atm (Figure 2E).The final result demonstrated complete restoration of flow with TIMI grade III in all vessels—left main, LAD, and left circumflex—with no residual stenosis in the LMCA (Figures 2F,G). Critically, the ostium of the LCX remained widely patent with preserved TIMI grade III flow, indicating no significant compromise from plaque shift or carina displacement (Figures 2F,G). Given this optimal angiographic result and preserved LCX flow, further intervention such as kissing balloon inflation was deemed unnecessary. Intravascular imaging was not performed given the urgent clinical scenario, which represents a limitation of our procedural approach.

### Follow-up and outcomes

**Figure 3:**
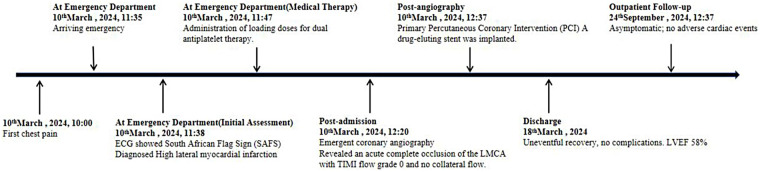
The patient's timeline. ECG:Electrocardiogram, LMCA, Left Main Coronary Artery, TIMI, Thrombolysis in Myocardial Infarction.

A repeat hs-cTnI measurement 4 h after successful PCI was 9.2 ng/mL, in line with a significant acute myocardial infarction and the early post-reperfusion phase.The medication regimen included 75 mg of aspirin daily, 90 mg of ticagrelor twice daily, 20 mg of atorvastatin nightly, 10 mg of ezetimibe nightly, 23.75 mg of extended-release metoprolol daily, and 40 mg of telmisartan daily. Echocardiography showed a left ventricular ejection fraction (LVEF) of 58%. Fortunately, the patient was discharged one week later without heart failure, malignant arrhythmias, or mechanical complications. Follow-up assessments at one and six months post-discharge were uneventful. The patient reported no angina or dyspnea (Canadian Cardiovascular Society (CCS) class I, New York Heart Association (NYHA) class I). A follow-up echocardiogram at six months showed preserved left ventricular function (LVEF 60%). She remained compliant with the dual antiplatelet therapy (aspirin and ticagrelor) along with other guideline-directed medical therapy (Figure 3 Timeline).

## Discussion

The South African Flag Sign (SAFS)—ST-segment elevation in leads I, aVL, and V2 with reciprocal ST-depression in lead III (1–3)—is a well-established electrocardiographic pattern that serves as a reliable marker for acute high lateral myocardial ischemia, most commonly attributed to occlusion of the first diagonal branch (D1) of the left anterior descending artery. In clinical practice, recognition of SAFS typically directs the interventionalist's attention toward D1 as the presumed culprit vessel. However, the electrocardiogram fundamentally maps ischemic myocardial territories rather than individual coronary arteries, and the same surface pattern can theoretically arise whenever the high lateral and anteroseptal walls are preferentially affected, regardless of the specific site of occlusion. Our case illustrates this principle in dramatic fashion: SAFS was the presenting ECG pattern in a patient with complete left main coronary artery (LMCA) occlusion—a life-threatening condition whose typical electrocardiographic signature is markedly different from the pattern observed here. This discordance between ECG prediction and angiographic reality underscores a clinically important diagnostic pitfall and warrants a systematic comparison of the ECG features associated with anatomically related culprit vessels.

The ECG manifestations of acute coronary occlusion vary substantially depending on the culprit vessel, yet considerable overlap exists among anatomically proximate locations. Acute total LMCA occlusion (ATOLMA) produces three principal ECG patterns: (1) extensive anterior and lateral ST-elevation (STEMI pattern) (4); (2) diffuse ST-depression across multiple leads with ST-elevation confined to aVR and/or V1, which is considered the most characteristic and prognostically ominous pattern (9, 10); and (3) a non-STEMI pattern with subtle or dynamic repolarization changes (11, 12). The ECG variability in LMCA occlusion reflects the vast territory at risk (>75% of the left ventricle), where opposing electrical vectors from simultaneously ischemic walls may partially cancel each other, and the presence of right coronary dominance and collateral circulation are key determinants of both the ECG manifestation and survival (12). Proximal LAD occlusion typically produces anterior precordial ST-elevation (V1–V4) with reciprocal inferior depression (13, 14); its important equivalent, de Winter syndrome—upsloping ST-depression with tall symmetric T-waves in V1–V6 without ST-elevation—accounts for approximately 2% of cases and requires emergent reperfusion (15–17). Isolated D1 occlusion generates the classic SAFS, with the combination of V2 ST-elevation and lead III ST-depression ≥2 mm carrying a positive predictive value of 98% for a large diagonal culprit. Ramus intermedius occlusion can produce an ECG pattern indistinguishable from D1 occlusion, because this trifurcation branch may perfuse a territory overlapping with the diagonal branches; the SAFS in this context reflects the ischemic region rather than a specific culprit vessel (18, 19). These overlapping patterns highlight the fundamental limitation of surface ECG localization: it identifies the territory at risk, not the artery responsible. To place our case in the broader literature context, Table 1 summarizes all published cases reporting SAFS with non-first diagonal branch culprit lesions (18–24). Notably, none of the previously reported cases involved complete LMCA occlusion, making the present case, to the best of our knowledge, the first reported association of SAFS with complete LMCA occlusion—though we acknowledge that atypical ECG patterns in LMCA occlusion have been previously described in other contexts.

**Table 1:** Summary of published cases reporting the South African flag sign (SAFS) with non-first diagonal branch culprit lesions.

Author (Year)	Age/Sex	Culprit Vessel	Hemodynamic Status	Outcome
Swarath et al. (20)	47y/F	Proximal LAD 100% occlusion	Hypertensive, tachycardic, no shock	Successful primary PCI to LAD;LVEF 40%–45%; discharged on GDMT
Granata et al. (19, 21)	66y/M	Isolated subocclusive proximal RI	Stable	Primary PCI to RI; uneventful recovery
Andriyan et al. (18)	65y/M	Total occlusion of proximal RI; severe proximal LAD stenosis	Stable	Staged PCI to RI and LAD; good outcome
Zahdeh (22)	69 y/M	Thrombotic occlusion of D1; complex LAD stenosis	Stable	Delayed PCI to D1 and LAD; TIMI 3 flow achieved
Granata et al. (19, 21)	∼80 y/F	Subocclusive LAD–D1 bifurcation; critical mid-RCA stenosis	Stable	Primary PCI to LAD–D1; staged PCI to RCA; LVEF 39%
de Alencar et al. (23)	58 y/M	No culprit lesion;mild mid-LAD irregularity	Stable	No revascularization; SAFS pattern classified as false-positive
Rathinasamy et al. (24)	56 y/M	Giant IgG4-related LAD pseudoaneurysm	Stable	Surgical aneurysmetomy + CABG; histology confirmed IgG4-RD
Present case	69y/F	LMCA(total occlusion)	Stable	Successful PCI, LVEF 58%

CAD, coronary artery disease; D1, first diagonal branch; DES, drug-eluting stent; GDMT, guideline-directed medical therapy; LAD, left anterior descending artery; LCx, left circumflex artery; LVEF, left ventricular ejection fraction; PCI, percutaneous coronary intervention; PLV, posterior left ventricular artery; RI, ramus intermedius; SAFS, South African Flag Sign; TIMI, Thrombolysis In Myocardial Infarction.

Our case adds to this evolving understanding by demonstrating that SAFS—a pattern at the opposite end of the expected ECG spectrum from classic LMCA occlusion—can indeed occur with complete left main occlusion. Several pathophysiological factors may explain this atypical presentation. First, when LMCA occlusion produces simultaneous transmural ischemia across the entire left coronary distribution, the surface ECG represents the vector summation of all affected territories; opposing electrical forces from the anterior, lateral, and inferior walls may partially cancel, allowing the territory with the greatest electrical mass—the high lateral and anteroseptal myocardium supplied by the proximal LAD and diagonal branches—to dominate the surface recording. Second, right coronary dominance in our patient limited the inferior wall's contribution to the overall electrical vector. Third, the complete absence of collateral circulation produced severe but regionally heterogeneous ischemia, further favoring the high lateral electrical signature. These factors collectively produced a SAFS pattern that mimicked isolated D1 or ramus intermedius occlusion. This observation does not redefine the diagnostic utility of SAFS but rather broadens its differential diagnosis to include, in rare instances, proximal LMCA occlusion. For clinical practice, the key message is that SAFS should prompt urgent angiographic evaluation without premature assumption of the culprit vessel, particularly when clinical features—such as hemodynamic instability, extensive wall motion abnormalities, or biomarker elevations disproportionate to the apparent ECG territory—suggest a more proximal or extensive lesion. It is important to note that this is a single case report, and the proposed pathophysiological mechanisms—while plausible—remain speculative and should not be extrapolated beyond this individual observation; systematic studies and further case accumulation are needed to validate whether specific clinical or ECG features can reliably distinguish LMCA-associated SAFS from its more common D1-related counterpart.

## Patient Perspective

“I suddenly felt a severe, crushing pain in my chest. I was terrified and thought I might not survive. In the Pingyuan County People's Hospital emergency department, everything moved very quickly. The doctors explained that the main artery to my heart was completely blocked and needed immediate surgery. The procedure was performed there by Dr. Zhong Fangming from Meizhou People's Hospital.The procedure was successful, and the pain resolved afterward. During my hospital stay, the team thoroughly explained my new medications and the importance of follow-up. Now, six months later, I feel like myself again with no chest pain. I am deeply grateful to the cardiology team at Meizhou People's Hospital for saving my life. I hope that sharing my story can help doctors recognize similar cases and improve care for other patients”.

## Conclusion

This unusual case highlights that the classic South African Flag Sign (SAFS), while typically indicative of a first diagonal branch occlusion, may exceptionally herald a life-threatening occlusion of the left main coronary artery. This observation underscores the critical importance of maintaining a high index of suspicion for more proximal and severe coronary pathology when encountering SAFS on ECG, prompting urgent angiographic evaluation to ensure appropriate and timely management.

## Preprint Statement

Preprint Declaration: An earlier version of this work was posted on Research Square (DOI: https://doi.org/10.21203/rs.3.rs-5419500/v1) and has since been withdrawn. The present manuscript represents a substantially revised and updated version.

## Data Availability

The original contributions presented in the study are included in the article/Supplementary Material, further inquiries can be directed to the corresponding author/s.
